# Dose- and time-dependent tolerability and efficacy of organo-osmium complex FY26 and its tissue pharmacokinetics in hepatocarcinoma-bearing mice

**DOI:** 10.1093/mtomcs/mfaa003

**Published:** 2020-12-11

**Authors:** Swati A Kumar, Russell J Needham, Kristin Abraham, Hannah E Bridgewater, Lauren A Garbutt, Helena Xandri-Monje, Robert Dallmann, Sebastien Perrier, Peter J Sadler, Francis Lévi

**Affiliations:** Chronotherapy Team, Division of Biomedical Sciences, Warwick Medical School, University of Warwick, Gibbett Hill Road, Coventry CV4 7AL, UK; Department of Chemistry, University of Warwick, Coventry CV4 7AL, UK; Chronotherapy Team, Division of Biomedical Sciences, Warwick Medical School, University of Warwick, Gibbett Hill Road, Coventry CV4 7AL, UK; Department of Chemistry, University of Warwick, Coventry CV4 7AL, UK; Chronotherapy Team, Division of Biomedical Sciences, Warwick Medical School, University of Warwick, Gibbett Hill Road, Coventry CV4 7AL, UK; Chronotherapy Team, Division of Biomedical Sciences, Warwick Medical School, University of Warwick, Gibbett Hill Road, Coventry CV4 7AL, UK; Chronotherapy Team, Division of Biomedical Sciences, Warwick Medical School, University of Warwick, Gibbett Hill Road, Coventry CV4 7AL, UK; Department of Chemistry, University of Warwick, Coventry CV4 7AL, UK; Department of Chemistry, University of Warwick, Coventry CV4 7AL, UK; Chronotherapy Team, Division of Biomedical Sciences, Warwick Medical School, University of Warwick, Gibbett Hill Road, Coventry CV4 7AL, UK; UPR ‘Chronotherapy, Cancers and Transplantation’, Faculty of Medicine, Paris Saclay University, Campus CNRS, 7 rue Guy Moquet, 94800 Villejuif, France; Hepato-Biliary Center, Paul Brousse Hospital, Assistance Publique-Hopitaux de Paris (AP-HP), 12–14 Avenue Paul-Vaillant Couturier, 94800 Villejuif, France

## Abstract

The organo-osmium complex [Os^II^(ɳ^6^-*p*-cym)(PhAzPy-NMe_2_)I]^+^ (FY26) exhibits promising *in vitro* antitumour activity against mouse hepatocarcinoma Hepa1–6 and other mouse or human cancer cell lines. Here, we drastically enhance water solubility of FY26 through the replacement of the PF_6_^−^ counter-anion with chloride using a novel synthesis method. FY26⋅PF_6_ and FY26⋅Cl displayed similar *in vitro* cytotoxicity in two cancer cell models. We then show the moderate and late anticancer efficacy of FY26⋅PF_6_ and FY26⋅Cl in a subcutaneous murine hepatocarcinoma mouse model. Both efficacy and tolerability varied according to FY26 circadian dosing time in hepatocarcinoma tumour-bearing mice. Tumour and liver uptake of the drug were determined over 48 h following FY26⋅Cl administration at Zeitgeber time 6 (ZT6), when the drug is least toxic (in the middle of the light span when mice are resting). Our studies suggest the need to administer protracted low doses of FY26 at ZT6 in order to optimize its delivery schedule, for example through the use of chrono-releasing nanoparticles.

## Graphical Abstract

**Graphical Abstract:**
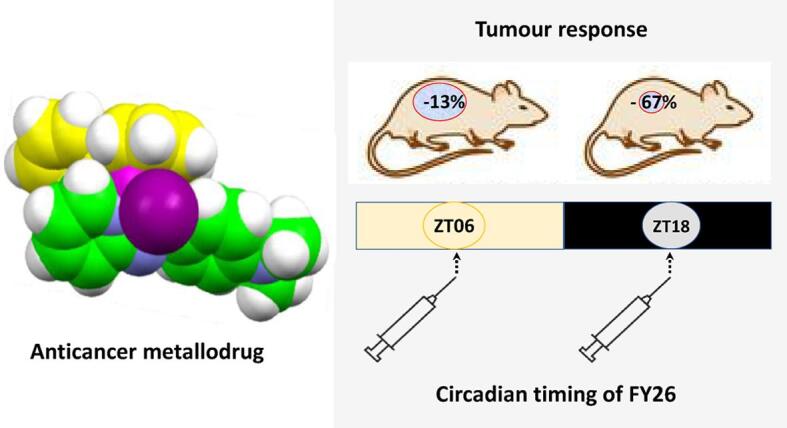
*In vivo* efficacy and tolerability of this inert organo-osmium anticancer complex are dependent on the time of administration (day versus night), consistent with preferential activation at specific stages of the circadian cycle.

## Significance to metallomics

Metallodrugs differ from most organic drugs in that they are prodrugs, which undergo transformation by ligand substitution and redox reactions before reaching target sites. Here, we study the *in vitro* and *in vivo* efficacy of an organo-osmium anticancer complex FY26—a relatively inert complex activated in cancer cells by attack on its azo bond by the tripeptide glutathione. We find that the efficacy and tolerability of FY26⋅PF_6_ or FY26⋅Cl are dependent on the time of administration, consistent with a role for the circadian clock in controlling expression of genes involved in glutathione synthesis.

## Introduction

There is a need for anticancer drugs with new mechanisms of action to complement platinum drugs in the clinic, reduce side effects, and overcome emerging platinum resistance. Complexes of ruthenium have been investigated in clinical trials,^1,2^ and compounds of heavier congener osmium are being explored.^3,4^ Although in the same periodic group, and often possessing complexes of similar composition (oxidation state, ligand set, size, and geometry), the reactivity of ruthenium and osmium complexes can differ markedly. Notably, organo-Os(II) arene complexes hydrolyse much more slowly and bound water ligands are more acidic.^5^

Numerous experimental and clinical studies have highlighted the relevance of circadian timing in drug administration for the optimization of pharmacological effects.^6,7^ For instance, dosing an anticancer medication at a specific time with reference to circadian rhythms can increase or decrease both efficacy and tolerability.^8–10^ As demonstrated for 28 anticancer agents belonging to various classes, the administration of a drug at the circadian time when it is best tolerated often achieves best antitumour activity.^6,7,9,10^ Here, we sought the relevance of this chronopharmacological principle along preclinical developmental stages of organo-Os complexes.

**Fig. 1:**
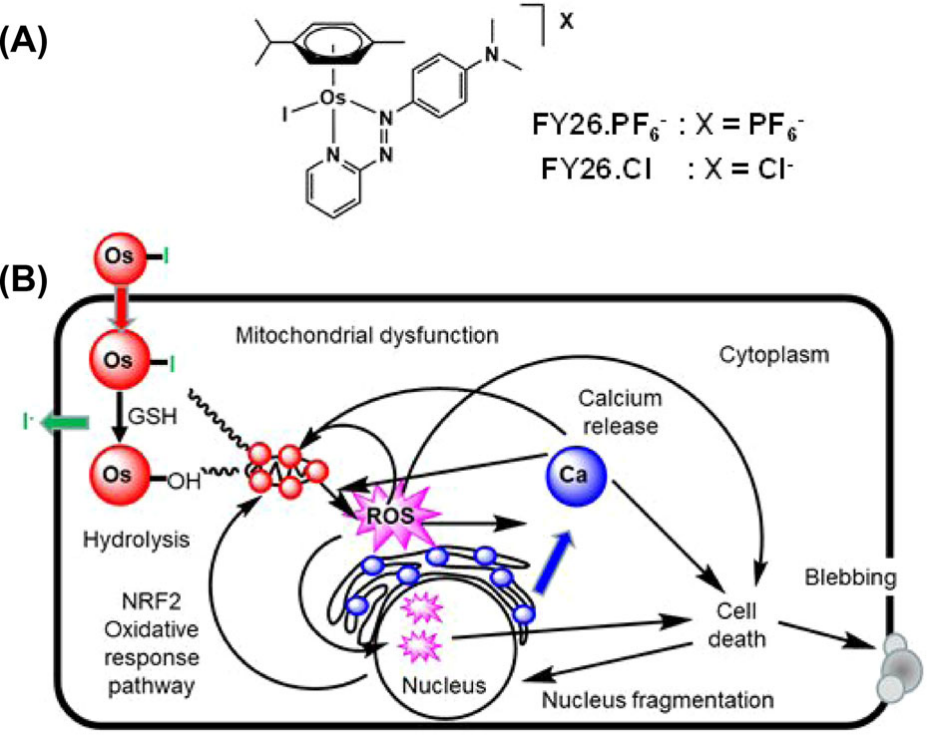
FY26 and its mechanism of action. (A) Structure of the organo-osmium azopyridine complex FY26 and abbreviations used for the PF_6_^−^ and Cl^−^ salts. (B) Proposed biomolecular mechanism for the cytotoxicity of FY26 involving intracellular activation by GSH-induced hydrolysis, calcium mobilization, mitochondrial dysfunction, ROS generation, and cell death.^20^

Organometallic half-sandwich Os(II) arene complexes with phenylazopyridine (PhAzPy) bidentate ligands show promising anticancer activity *in vitro* against a variety of cancer cell lines,^11,12^ and also *in vivo*,^13^ in contrast to less-active organo-Os(II) drugs with *N,N*-chelated diamine or *N,O*-chelated picolinate ligands, which rely primarily on the dissociation of labile ligands followed by DNA binding.^14,15^ Iodido Os(II) PhAzPy complexes are considerably more stable in aqueous media and exhibit a mechanism of activity involving production of intracellular reactive oxygen species (ROS). These inert complexes are activated in cancer cells by attack of glutathione on the azo bond of the chelated PhAzPy ligand.^16,17^ A key complex, FY26⋅PF_6_ ([Os^II^(ɳ^6^-*p*-cym)(PhAzPy-NMe_2_)I]PF_6_), is on average 49 times more active than cisplatin in the 809 cancer cell line screen of the Sanger Institute,^18^ and capable of delaying the growth of HCT-116 human colon cancer xenografts in mice.^13^ Furthermore, it is up to 64 times more selective towards A2780 ovarian cancer cells over MRC-5 fibroblasts when applied synergistically with l-buthionine sulfoximine (l-BSO).^19^ The structure of FY26 and its biomolecular mechanism of action are shown in Fig. 1.

*In vitro* experiments have also demonstrated time-dependent efficacy of FY26⋅PF_6_ against both mouse Hepa1–6 hepatocarcinoma and human HCT-116 colon carcinoma in cell cultures.^21^ More specifically, the IC_50_ of FY26⋅PF_6_ varied by 40% according to the timing of *in vitro* drug exposure of cultured Hepa1–6 cells, following the synchronization of their circadian clocks with a 24-h periodic temperature cycle.^21,22^ The least toxic timing for FY26⋅PF_6_ administration was near the minimum of the sinusoidal temperature cycle that synchronizes the genetic circadian clocks of the cells in the Petri dishes over the 24 h. FY26⋅PF_6_ chronopharmacology mechanisms depend upon clock gene Bmal1 proficiency, and involve circadian-dependent FY26 uptake and possibly efflux.^2^ Moreover, the toxicity of FY26⋅PF_6_ also varies according to dosing time in mice.^22^ The best-tolerated time for FY26 in mice following dosing is near the middle of the light span, which corresponds to the low point in their endogenous circadian rhythm in core body temperature. Strikingly, the circadian rhythm in FY26⋅PF_6_ toxicity for Hepa1–6 cells and for mice differs from those in oxaliplatin toxicity by several hours, hence further highlighting the pharmacological differences between these organo-osmium complexes and platinum metallodrugs on the temporal timescale.^22^

Here, we aimed to optimize the chemical solubility of FY26 prior to the determination of FY26 efficacy and tolerability in Hepa1–6 tumour-bearing mice. We further obtained insight into its tissue uptake, so as to optimize its dosing schedule, in terms of both the frequency of administration and circadian timing.

## Materials and methods

### FY26 synthesis and characterization

FY26⋅PF_6_ was synthesized and characterized as previously reported^12^, however, it was poorly water-soluble. We previously reported that the anion (X, Fig. 1A) has a major effect on aqueous solubility for organo-osmium complexes.^23^ Here, we synthesized FY26 as a chloride salt (FY26⋅Cl) instead of PF_6_. Furthermore, the route by which FY26 is synthesized was improved upon, now involving a two-step one-pot synthesis from [Os(η^6^-*p*-cym)Cl_2_]_2_. FY26 was first isolated as an iodide salt (FY26⋅I), which is an important purification step prior to anion exchange with chloride. The anion exchange was conducted by passing FY26⋅I through Dowex^®^ 1X4 chloride form resin, yielding FY26⋅Cl.

### Synthesis of FY26⋅Cl

[Os(η^6^-*p*-cym)Cl_2_]_2_ (4.0 g, 5.06 mmol) was dissolved in methanol (50 ml) and heated to 80°C. A solution of potassium iodide (84.0 g, 505.91 mmol) in water (50 ml) was added and the mixture stirred for 5 min. A solution of *N,N*-dimethyl-4-(2-pyridylazo)aniline (2.4 g, 10.62 mmol) in methanol (50 ml) was added dropwise and the mixture was stirred for 1 h at 80°C, and left stirring for 18 h at ambient temperature. The majority of methanol was removed under reduced pressure using a rotavap and the product was extracted with dichloromethane (100 ml), and washed with water (100 ml). More product was extracted from the aqueous layer with dichloromethane washings (4 × 50 ml). The combined dichloromethane extracts were vacuum filtered to remove a black impurity, and the filtrate was dried under reduced pressure. The solids were fully redissolved in a mixture of ethanol (200 ml) and dichloromethane (50 ml). The volume of the solution was reduced using a rotavap to ∼40 ml and a precipitate formed, and then the mixture was placed in a freezer (−20°C) overnight. The intermediate product (FY26⋅I) was collected via vacuum filtration and washed with ice-cold ethanol (∼10 ml), and then diethyl ether (6 × 50 ml). The washes were repeated once more. FY26 was collected, analysed by high-performance liquid chromatography (HPLC; 99.4% pure; C18 column: 250 mm × 4.6 mm and pore size = 5 µm; mobile phase: acetonitrile/water + 0.1% trifluoroacetic acid), and dissolved in methanol (100–150 ml).

A chromatography column (250 ml) containing Dowex^®^ 1X4 chloride form resin was prepared (Sigma-Aldrich, Dorset, UK). The resin was flushed with aqueous sodium chloride solution (0.1 g/ml, 0.8 l), water (1.0 l), methanol:water (1:1, v/v, 0.8 l), and then methanol (0.4 l). The FY26⋅I methanol solution was passed through the column using methanol as an eluent. The dark blue complex was collected and any other fractions were discarded. The extract was evaporated to dryness under reduced pressure, and the solids were redissolved in a minimum volume of dichloromethane. This was dried once more under reduced pressure, forming a shiny flaky dark blue amorphous solid, which was isolated and dried overnight in a vacuum desiccator. Yield: 7.09 g, 98%. ^1^H NMR ((CD_3_)CO): *δ* 9.91–9.93 (m, 1H), 8.56–8.58 (m, 1H), 8.15–8.22 (m, 3H), 7.56–7.60 (m, 1H), 6.93–6.95 (m, 2H), 6.81–6.82 (m, 1H), 6.68–6.70 (m, 1H), 6.34–6.36 (m, 1H), 6.28–6.29 (m, 1H), 3.35 (s, 6H), 2.77 (s, 3H), 2.52 (sept., 1H, *J* = 6.9 Hz), 0.97 (d, 3H, *J* = 6.9 Hz), and 0.91 (d, 3H, *J* = 6.9 Hz). ESI-MS calculated for C_23_H_28_IN_4_Os^+^: *m/z* 679.1. Found: 679.1. CHN and Cl analysis: found: C, 38.99%; H, 4.57%; N, 7.22%; Cl, 4.69%. Calculated for C_23_H_28_ClIN_4_Os: C, 38.74%; H, 3.96%; N, 7.86%; Cl, 4.97%. HPLC purity: 99.4%.

### Comparative solubility study

One to five milligrams of FY26⋅PF_6_ or FY26⋅I, and 180 mg of FY26⋅Cl were dispensed into Falcon tubes in triplicates, then 1 ml of doubly deionized water (DDIW) was added. The Falcon tubes were placed on a Vibrax VXB basic shaker (Sigma-Aldrich, Dorset, UK) for 24 h at a speed of 500 × *g*/min (ambient temperature 18–20°C), and were checked at intervals (further solids were added if total saturation appeared to occur). The samples were filtered through syringe filters (PTFE, 0.45 µm, 4 mm) to remove any undissolved solids. The concentrations of Os in the solutions were measured using inductively coupled plasma-optical emission spectroscopy (ICP-OES) (Agilent, Stockport, UK).

### Separation of FY26 enantiomers via chiral HPLC

A solution of FY26 in EtOH (1 mg/ml) was prepared and filtered through a syringe filter (PTFE, 4 mm × 0.45 μm). Preparative chiral HPLC was carried out using an Agilent Technologies 1200 series HPLC instrument with a VWD and 1 ml loop. The instrument was fitted with a DAICEL Group (Chiral Technologies Europe) Chiralpak IC cellulose-based semi-preparative column (250 mm × 10 mm, 5 µm pore size) and a guard column (same brand and stationary phase: 10 mm × 20 mm, 5 µm pore size). The mobile phase consisted of EtOH:*n*-heptane (1:1, v/v), with 0.5% trimethylamine (TMA, v/v) and 0.3% trifluoroacetic acid (v/v) additives. The mobile phase was isocratic with a flow rate of 3 ml/min. Aliquots of 40× 500 μl were injected manually with a sample run time of 30 min (wavelength of detection = 254 nm). Two distinct peaks were observed with retention times of 17.9 min (enantiomer 1) and 20.3 min (enantiomer 2), and collected manually. Combined fractions were evaporated to dryness, redissolved in MeCN:H_2_O (1:9, v/v, 10 ml), and purified by preparative reverse-phase HPLC to remove excess TMA and its salts. Excess NH_4_PF_6_ (∼100 mol equiv.) was combined with each enantiomer and the HPLC solvents were removed under reduced pressure. The enantiomers were dissolved in dichloromethane (5 ml), filtered, dried under vacuum, and stored in a freezer (−20°C).

### Biological assays

An *in vitro* study first investigated the relevance of iodide or chloride anion for the maximum solubility and cytotoxicity of FY26. Five *in vivo* experiments (Experiments 1–5) were performed involving a total of 170 mice in order to determine (i) the antitumour efficacy of FY26 against measurable hepatocarcinoma (Hepa1–6-*Per2-luc*), (ii) the possible limiting toxicity and the influence of dosing time, and (iii) the relationship between dose level and tumour and liver drug uptake (Table 1).

**Table 1.** Endpoints and main characteristics of the five mouse experiments

Experiment no.	Endpoints	Daily FY26 dose (mg/kg) and ZT	No. of injections (no. of days between injections)	Cumulative dose given (mg/kg)	Treatment duration (days)	Dose intensity (mg/kg/day)	No. of mice, strain (no. of treated/controls)
1	Tumour and body weight changes	40, 60, or 80 (ZT6)	6 (2)	240–480	12	20–40	18, CD1-*Foxn1^nu^* (15/3)
2	Tumour and body weight changes	50 (ZT6 or ZT18)	6 (2)	300	12	25	20, CD1-*Foxn1^nu^* (12/8)
3	Tumour and body weight changes	20 (ZT6)	3 (2–3)	60	5	12	36, C57Bl/6 (20/16)
4	Tumour and body weight changes	15 (ZT6 or ZT18)	4 (3–4)	60	10	6	30, C57Bl/6 (20/10)
5	[Os] (ng/g) in tumour, liver, and five organs; tumour and body weights	0, 5, 10, or 20 (ZT6)	1 (tissue samples at 6, 24, and 48 h)	5–20	1	NA	66, C57Bl/6 (57/9)
All		5–80	1–6 (2–4)	5–480	1–12	1–40	170 (124/46)

FY26⋅PF_6_ was used in Experiments 1 and 2, while FY26⋅Cl was administered in Experiments 3–5.

### Cell culture

Dulbecco's modified Eagle's medium (DMEM) cell culture media, penicillin/streptomycin, foetal bovine serum, l-glutamine, phosphate-buffered saline (PBS) solution, and trypsin/EDTA were all purchased from Thermo Fisher (Loughborough, UK). DMEM was supplemented with 10% foetal bovine serum, 1% penicillin/streptomycin, and 2 mM l-glutamine. Cancer cell lines (A2780 and A549) were purchased from the European Collection of Cell Cultures (Salisbury, UK). Cell cultures were grown in T-75 or T-175 culture flasks as adherent monolayers. The cells were passaged two to three times per week whenever confluence reached 80–90%, using 0.25% trypsin/EDTA. Cell cultures were stored in an incubator at 37°C with a 5% CO_2_ humidified atmosphere.

### *In vitro* growth inhibition assay

Five thousand A2780 or A549 cells were seeded per well in 96-well plates. The cells were pre-incubated in drug-free media at 37°C for 48 h before adding a range of concentrations (0.01–100 µM) of the complexes to be tested. Typically, the complex was dissolved in dimethyl sulfoxide (DMSO) at 2 mM concentration, and then diluted with cell culture medium to give a 100 µM stock solution with 5% (v/v) DMSO. Cells were exposed to complexes for 24 h at 37°C. The supernatants were removed by suction and each well was washed with PBS. The cells were allowed to recover for 72 h in a drug-free medium at 37°C. The SRB assay was used to determine cell viability.^24^ Absorbance measurements of the solubilized dye (Thermo Scientific Multiskan FC with SkanIt software 4.1 for Windows using a 470 nm filter) allowed the determination of viable treated cells compared to untreated controls. IC_50_ values (the concentration at which 50% cell death occurs) were determined as triplicates of duplicates for each complex. ICP-OES was used to determine the osmium concentrations of the initial stock solutions of the complexes, and hence the IC_50_ values.

### ICP-OES

Analyses of FY26 solutions were carried out on a Perkin-Elmer (optical emission spectrometer) Optima 5300 DV instrument. All samples and standards were prepared fresh on the day in DDIW with distilled HNO_3_ (3.2%), thiourea (5 mM), and ascorbic acid (0.1 g/l). The osmium standard was diluted to the following concentrations for the calibration curve: 0, 25, 50, 100, 200, 400, 600, 800, and 1000 ppb. The samples were diluted by serial dilutions of typically 1 in 10 until their concentrations fitted within the calibration range and % total dissolved solids were below 0.2%. Samples were prepared in triplicates and the Os optical emissions at 228.226 and 225.585 nm were detected and integrated.

### Animals

Male CD1-*Foxn1^nu^* or C57Bl/6 (B6) mice aged 5–10 weeks (Janvier, Le Genest St Isle, France) were housed in groups of three to four mice per cage in temperature-controlled (23 ± 1°C), ventilated (100 ± 10 l/min), and light-tight chronobiological animal facilities (Eurobioconcept, Bonneuil s/Marne, France). They were synchronized with an alternation of 12 h of light (L) and 12 h of darkness (D) (LD12:12), with light starting at 07:00, and had *ad libitum* access to food and water throughout all the experiments. In line with convention, onset of light and that of darkness are referred to as Zeitgeber time 0 (ZT0) and ZT12, respectively. After one or more weeks on this regimen and on the day prior to tumour inoculation, both flanks of each B6 mouse were shaved using clippers.

### Tumour inoculation and growth assessment

Mouse hepatocarcinoma (Hepa1–6, ATCC^®^ CRL-1830™) was kindly provided by S. Dulong (INSERM U935, Villejuif, France), following their stable transfection with the *Per2-luc* [pLV7-*Bsd*-P(*Per2*)-d*Luc*] reporter constructs within the C5SYS ERASYSBIO+ project (FP7). Mouse Hepa1–6-*Per2-luc* cells were cultured in 15-cm Petri dishes until they reached 80% confluency. On the day of tumour inoculation, the cells were trypsinized and resuspended at a concentration of 10^6^ cells/100 µl in PBS solution alone (Experiments 1 and 2) or with 1:1 MatriGel™ (Corning, Flintshire, UK) (Experiments 3–5) and kept on ice until injected.

The mice, aged 8–10 weeks, were anesthetized under isoflurane and Hepa1–6-*Per2-luc* cells were injected subcutaneously in both postero-lateral flanks of each mouse. Mice were then inspected and weighed daily until experiment completion. Tumour sites were inspected daily until tumours became palpable and amenable to daily measurements of perpendicular diameters using callipers. Measurements were always taken at the same circadian time.

Mean daily tumour weight per mouse was calculated as the average volume of the right and left flank tumours according to (*L* × *W*^2^)/2, where *L* is the larger and *W* the smaller perpendicular diameter, and 1 cm^3^ corresponds to 1 g. Mice with an estimated tumour weight exceeding 10% of their body weight or those in poor general condition or loosing >15% of their starting body weight were euthanized, in compliance with our Home Office Project Licence specifications approved by the Animals in Science Committee and Animal Welfare and Ethical Review Body.

### Experimental objectives and design

All experiments were performed in compliance with the UK relevant laws and institutional guidelines, and were approved by the Animals in Science Committee and the Animal Welfare and Ethical Review Body of the University of Warwick.

Experiment 1 aimed at the determination of a therapeutic dose of the less soluble FY26⋅PF_6_ formulation. Hepa1–6-bearing nude mice were randomly assigned to be injected intraperitoneally (ip) with vehicle (controls), or 40, 60, or 80 mg/kg/injection of FY26⋅PF_6_ at ZT6 (in the middle of the light span) every other day for 12 days (Table 1).

Experiment 2 aimed at assessing whether FY26⋅PF_6_ circadian timing would influence toxicity or efficacy in the nude mouse model. The animals were randomly assigned to receive ip vehicle or FY26⋅PF_6_ (50 mg/kg/injection) at ZT6 or at ZT18 (four controls and six treated mice per ZT) every other day for 11 days (six injections; cumulative dose, 300 mg/kg).

Experiment 3 aimed at assessing the efficacy and tolerability of the more soluble formulation of FY26⋅Cl in Hepa1–6-bearing C57Bl/6 mice. The mice were randomized to receive vehicle or FY26⋅Cl, at a cumulative dose of 60 mg/kg. Treated mice received ip injections of 20 mg/kg of FY26⋅Cl at ZT6 12, 14, and 17 days after tumour inoculation. Control mice received ip vehicle on the same days.

Experiment 4 aimed at confirming efficacy and optimizing timing and scheduling of the same cumulative dose of 60 mg/kg of FY26⋅Cl. Mice were randomized to receive either vehicle or FY26⋅Cl as four injections of 15 mg/kg at ZT6 or ZT18 on days 11, 14, 17, and 21.

Experiment 5 determined FY26⋅Cl uptake in mouse tumour and liver. Hepa1–6-bearing mice were randomly assigned to either treatment or control groups. They received a single ip injection of 5, 10, or 20 mg/kg of FY26⋅Cl or 0.1 M PBS (vehicle) at ZT6 (Table 1). The mice from pre-set subgroups were culled at 6, 24, or 48 h after injection. The subcutaneous tumours in both flanks, as well as liver, spleen, one kidney, an ileum, and a colon segment and brain were sampled and stored at −80°C.

### FY26 sample preparation

For Experiments 1 and 2, FY26⋅PF_6_ was diluted in the vehicle, composed of 1% Tween 80 (Sigma-Aldrich, Gillingham, UK), 5% DMSO (Corning, New York, USA) and 94% of 0.9% saline (Prep room, Life Sciences, University of Warwick) under laminar flow. FY26⋅PF_6_ concentration was confirmed with ICP-MS. For Experiments 3–5, using FY26⋅Cl, a stock solution of 50 mg/ml FY26⋅Cl was prepared in 0.9% saline on the day of injection and diluted as required; the vehicle consisted of 0.9% saline.

### Tissue sample preparation and chemical digestion

Liver tissue was homogenized by manual mechanical grinding (pestle and mortar) while frozen under liquid N_2_. Once thawed, ∼90–110 mg of homogenized tissue was pipetted into microwave reaction vessels (10 ml), and tetramethylammonium hydroxide (TMAH, 25% m/v, 2 ml) was added. Tumour tissue was not homogenized and instead digested as whole tissue. It was weighed directly into microwave vials and TMAH was added (100 µl per 5 mg tissue, minimum of 500 µl added). The samples were digested via microwave irradiation (CEM SP-D Discover^®^ microwave reactor, ≥300 W, ≥450 psi) at 200°C for 10 min. Digested samples were diluted in one part DDIW, and then centrifuged at 9000 rpm for 10 min at 20°C. The final analytical samples were prepared by a further 1 in 12.5 dilution in DDIW (total dilution of 1 in 25), ensuring a final TMAH concentration of 1% and total dissolved solids <0.1% and the total dissolved solids were <0.1%. TMAH digestion was selected to circumvent the formation of volatile Os species with nitric acid.

### ICP-MS analysis

Analyses of digested tissue samples were carried out on an Agilent Technologies ICP-MS 7900 spectrometer in a basic aqueous matrix of TMAH (1%, m/v). The osmium concentrations were determined via isotopic detection of ^189^Os in gas (He) and no-gas modes, and samples were referenced internally to ^100^Ru (10 ppb). A concentration calibration curve was produced by serial dilutions of an osmium standard (1000 ppm) in 1% TMAH: 0, 0.1, 0.25, 0.5, 1, 2.5, 5, 10, 25, 50, 100, 250, 500, and 1000 ppb.

### Statistical methods

Body weight data were expressed as % body weight measured on the first treatment day. Tumour weights for individually treated mice were expressed as % of the median control values, in order to determine % inhibition. Mean and standard error or median and interquartiles were computed for the different treatment groups and experimental conditions, i.e. drug timing and day of assessment. Groups were compared using parametric or non-parametric ANOVAs, respectively.

## Results

### Comparability of FY26⋅X maximum solubilities

**Fig. 2:**
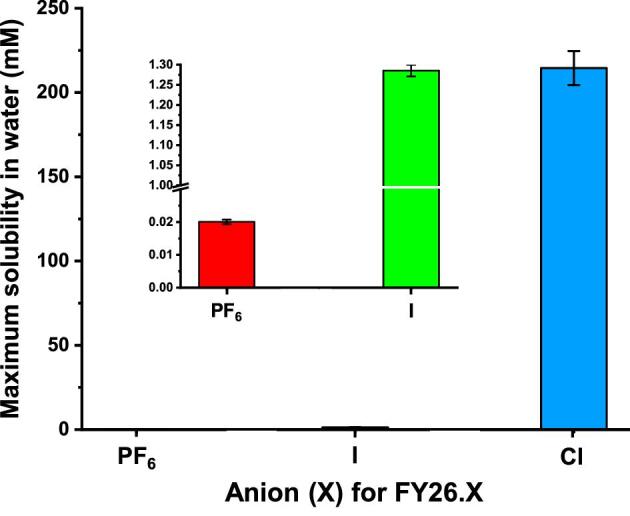
Bar graph of the dependence of the maximum solubilities of FY26⋅X on the anion X, in water (where X = PF_6_^−^, I^−^ or Cl^−^). Data are mean ± SD of *N* = 3, being 0.0201 (±0.0007), 1.29 (±0.1), and 215 (±10) mM for FY26⋅PF_6_, FY26⋅I, and FY26⋅Cl, respectively.

The maximum aqueous solubilities of FY26⋅X (where X = PF_6_, I, or Cl) were determined in DDIW. Their solubilities were measured as 0.0201 (±0.0007), 1.29 (±0.01), and 215 (±10) mM for X = PF_6_, I, and Cl, respectively. A dramatic 10,448-fold increase in solubility is observed by switching the counter-anion from PF_6_ to Cl (Fig. 2).

### *In vitro* antiproliferative activity of FY26⋅X and its enantiomers

**Fig. 3:**
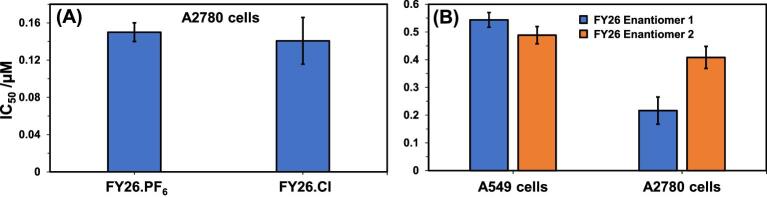
Bar graphs illustrating the *in vitro* anticancer efficacy of different forms of FY26. (A) Antiproliferative activities of FY26⋅PF_6_ and FY26⋅Cl against A2780 cancer cells. Data represent mean ± SD of *N* = 6. (B) Antiproliferative activities of the FY26 enantiomers against A2780 and A549 cancer cells. Mean ± SD of *N* = 6.

The antiproliferative activity of FY26⋅X was measured in A2780 ovarian cancer cells, where X, the counter-anion, was varied (PF_6_ or Cl, Fig. 3). The data confirm the lack of any statistically significant difference in anticancer activity between the new highly soluble formulation (X = Cl) and the older formulation (X = PF_6_). Furthermore, FY26 has a chiral metal centre and exists as a racemic mixture of two enantiomers as shown by its X-ray crystal structure.^25^ As previously reported for FY26⋅PF_6_,^18^ FY26⋅Cl similarly did not induce apoptosis on exposure in A2780 cells but did induce an increase in ROS (data not shown). Herein, the enantiomers were separated via chiral HPLC and individually tested against A2780 ovarian and A549 lung cancer cells (Fig. 3). The activity of both enantiomers was similar, although for A549 cells the IC_50_ of one was 1.8x that of the other.

### Dose and time dependences of FY26⋅PF_6_ effects in tumour-bearing nude mice

In Experiments 1 and 2, tumours grew exponentially up to a median value of ∼1.2 and ∼1 g, respectively, which was reached 14 days after inoculation. In Experiment 1, a dose response was documented for tumour inhibition, with efficacy ranking as highest for the dose of 80 mg/kg/injection, intermediate for that of 40 mg/kg/injection, and least for 60 mg/kg/injection.^22^ Body weight loss displayed a linear dose–response relation, with the higher dose being clearly too toxic since all the mice treated at this dose level were euthanized after up to five injections (cumulative dose of 400 mg/kg).^22^

**Fig. 4:**
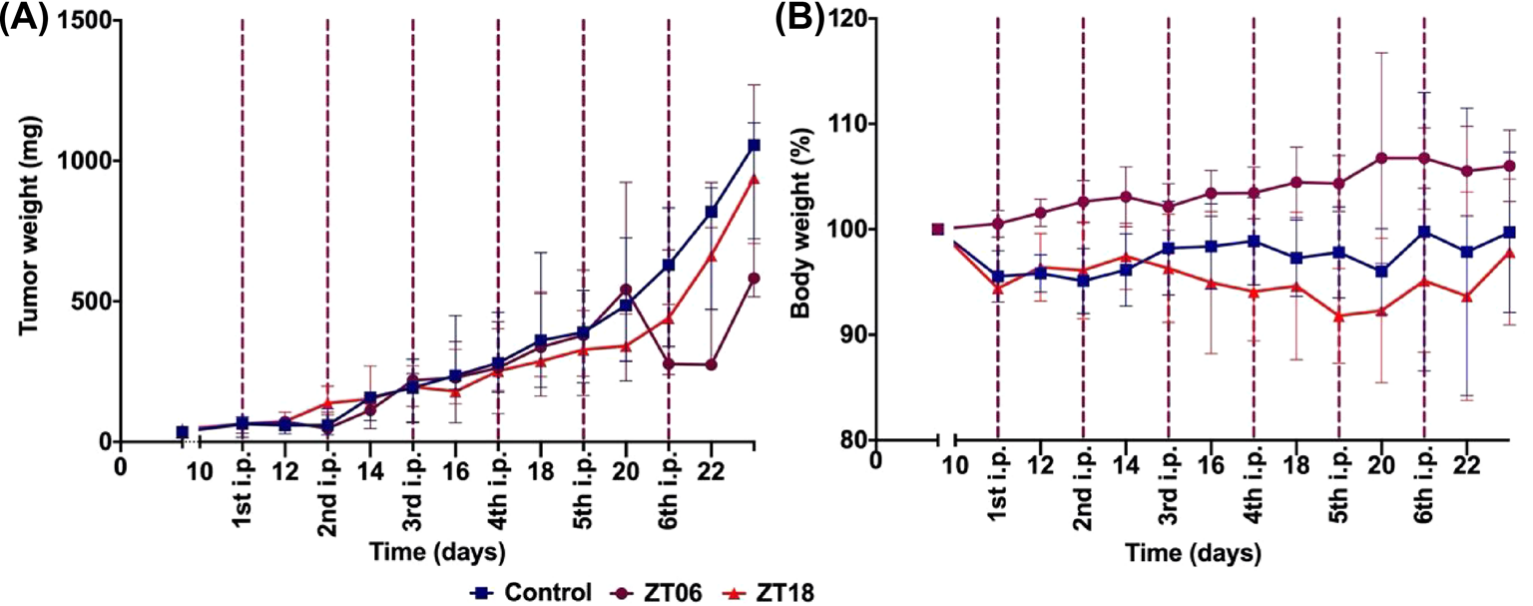
Dosing time effects of FY26 in Hepa1–6-bearing nude mice (Experiment 2). Effects of FY26⋅PF_6_ (50 mg/kg/injection) on tumour weights (A) and body-weight changes (B), according to injection timing (Zeitgeber time). Data shown in panels A and B are median ± interquartile range (IQR).

In Experiment 2, tumour growth slowed down transiently after the sixth FY26⋅PF_6_ dose, with a slightly better efficacy at ZT6 (Fig. 4A). Body-weight loss was less pronounced in the mice treated at ZT6 as compared to ZT18 (Fig. 4B).

### Schedule-dependent antitumour efficacy of FY26⋅Cl in C57Bl/6 mice

The distributions of mean tumour weights per mouse were similar in control and treatment groups both within and between experiments before FY26 treatment in Experiments 3 and 4. Indeed, no statistically significant difference was found for comparisons of treatment versus control groups (*P* = 0.843) or for that of Experiment 3 versus Experiment 4 (*P* = 0.671), and no interaction term was validated, with two-way ANOVA. Large interindividual variations were found, however, since the average tumour weight that was measured in each mouse varied by >10-fold in both control and treatment groups of each experiment.

**Fig. 5:**
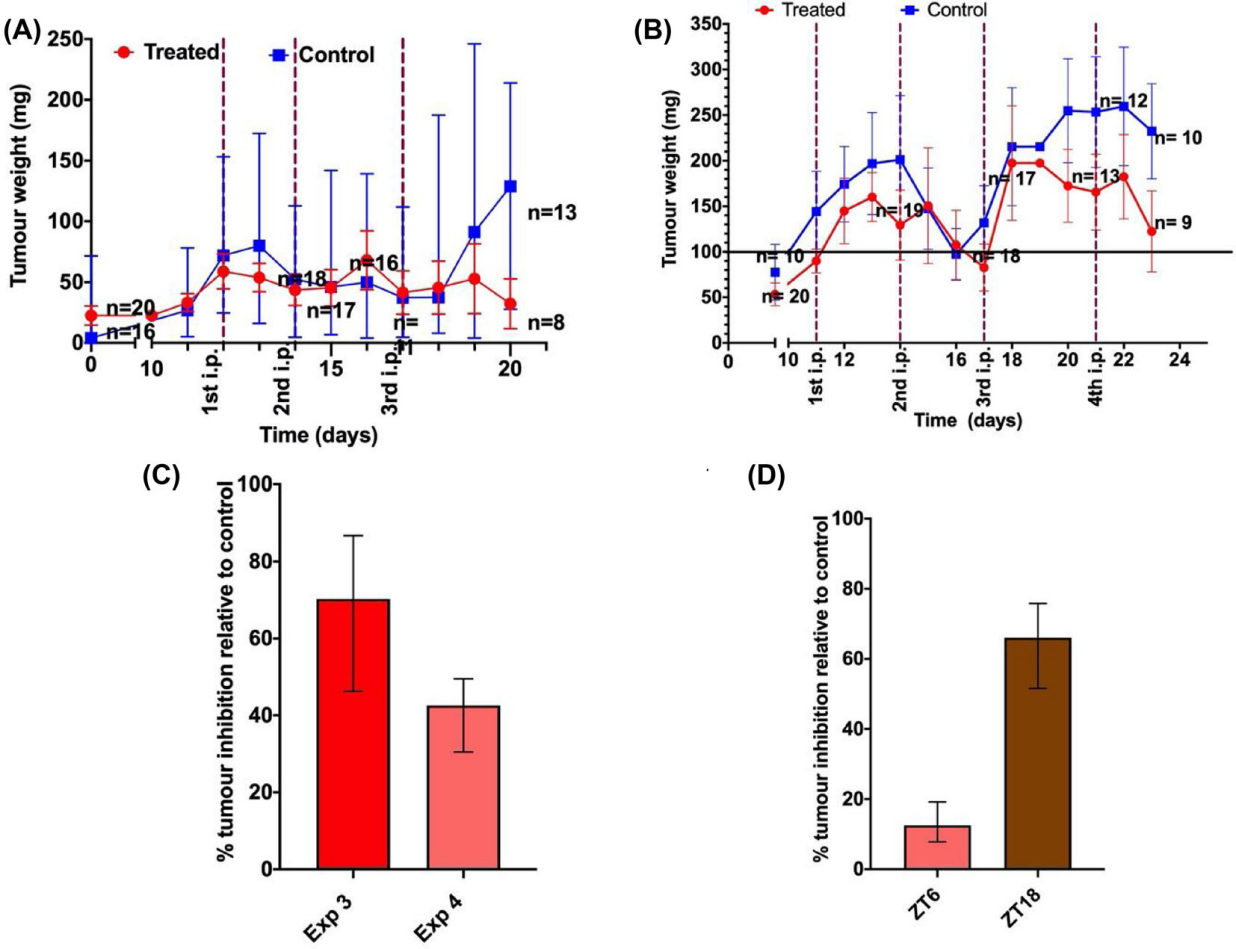
Antitumour efficacy of FY26⋅Cl in Hepa1–6-bearing C57Bl/6 mice. Growth curves of inoculated Hepa1–6-*Per2-luc* tumours in male C57Bl/6 mice treated with vehicle (control groups) or FY26⋅Cl (treatment groups). Median daily tumour volumes (±interquartiles) in treated mice or controls receiving vehicle. (A) Experiment 3, with mice in the treatment group with 20 mg/kg of FY26⋅Cl ip per injection × three injections. (B) Experiment 4, with mice in the treatment group dosed with 15 mg/kg of FY26⋅Cl ip per injection × four injections. (C) FY26⋅Cl induced tumour-weight (%) inhibition relative to median control tumour weight. (D) Tumour-weight inhibition (%) relative to median control tumour weight, according to FY26⋅Cl timing. Data shown in panels C and D are median ± IQR.

The tumour weights in controls nearly doubled over the 8–12 days following the first vehicle administration, but did not exceed 0.25 g, thus indicating a slower tumour growth in the C57B/L6 mice (Experiments 3 and 4) as compared to the CD1-*Foxn1^nu^* (Experiments 1 and 2). Spontaneous partial tumour regressions and regrowth were noticed. Tumours tended to be smaller in the treated mice throughout each experiment, and a clear antitumour effect of FY26⋅Cl was demonstrated 20 and 23 days after tumour inoculation in Experiments 3 and 4, respectively (Fig. 5). The treated mice in both experiments had then received a cumulative dose of 60 mg/kg over 5 days for Experiment 3, and over 10 days for Experiment 4. For Experiment 3, the median tumour weight was 30.2 mg for the treatment group and 128.8 mg for the control group on day 20 (Fig. 5A). In Experiment 4, these respective values were 122.3 mg (treatment groups ZT6 and ZT18) and 213.2 mg (control group) on day 23 (Fig. 5B). The higher dose intensity of FY26 was associated with a significantly larger tumour inhibition rate (Kolmogorov–Smirnov test, *P* = 0.0217). Thus, at experiment completion, the median percentage of FY26⋅Cl-induced tumour inhibition relative to controls was 76.5% in Experiment 3, where the drug was given at ZT6 only, and 42.6% in Experiment 4 irrespective of dosing time. At the end of Experiment 4, the percentage inhibition relative to control was 12.5% in the mice dosed with FY26⋅Cl at ZT6 and 66% in those treated at ZT18 (Kruskal–Wallis ANOVA, *P* = 0.022) (Fig. 5D). FY26 efficacy, however, could be determined only for those mice that survived the treatment toxicities.

### Circadian timing-dependent toxicity of FY26⋅Cl

Thus, at study completion, 13/20 controls in Experiment 3 and 10/10 controls in Experiment 4 were alive, representing an overall rate of 23/30 controls for both experiments (76.7%). Seven control mice (23.3%) had to be euthanized due to tumour progression. In contrast, severe clinical toxicity signs were observed that mandated euthanizing nearly half of the treated mice for ethical reasons. As a result, 8/16 (50%) and 9/20 treated mice (45%) were alive at completion of Experiments 3 and 4, respectively. In Experiment 4, overall survival was best following FY26 dosing at ZT6 as compared to ZT18 with respective survival rates of 60% versus 30% (Gehan–Breslow–Wilcoxon test, *P* = 0.0012). Body-weight loss, however, did not correlate with other signs of physical condition that triggered humane endpoints.

**Fig. 6:**
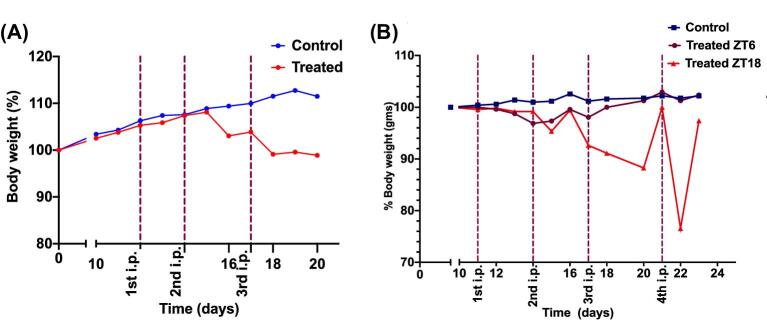
Relative changes in body weight following vehicle (control groups) or FY26⋅Cl (treatment groups) administration in Hepa1–6-*Per2-luc* tumour-bearing mice treated in two experiments. Median daily body weight (±interquartiles). (A) Experiment 3, mice in the treatment group receiving 20 mg/kg of FY26 ip per injection × three injections at ZT6. (B) Experiment 4, mice in the treatment group dosed with 15 mg/kg of FY26 ip per injection × four injections at ZT6 or ZT18.

Daily body weights increased gradually in controls over the respective treatment durations in each experiment. The FY26-treated mice displayed a modest yet consistent decrease in body weight as compared to pre-treatment values in both experiments. Body weight was primarily reduced after the second injection and then remained rather stable despite further FY26 administrations (Fig. 6).

Thus, the body weight changes showed a significant difference between the treatment and the control group after the third dose in each experiment. After a cumulative dose of 60 mg/kg of FY26⋅Cl, mice lost ∼10% (±3.2%) of their body weight before treatment. Body-weight loss from FY26 dosing was clearly worse at ZT18 as compared to ZT6 (Fig. 6B). Mann–Whitney test validated significant differences in body weight changes between controls and FY26-treated mice over each experiment duration irrespective of drug timing (*P* = 0.0017 and *P* = 0.0059 for Experiments 1 and 2, respectively), as well as for circadian timing effects (Dunnett's multiple comparison test, *P* < 0.0001).

### FY26 tumour and liver pharmacokinetic and pharmacodynamic correlations

**Fig. 7:**
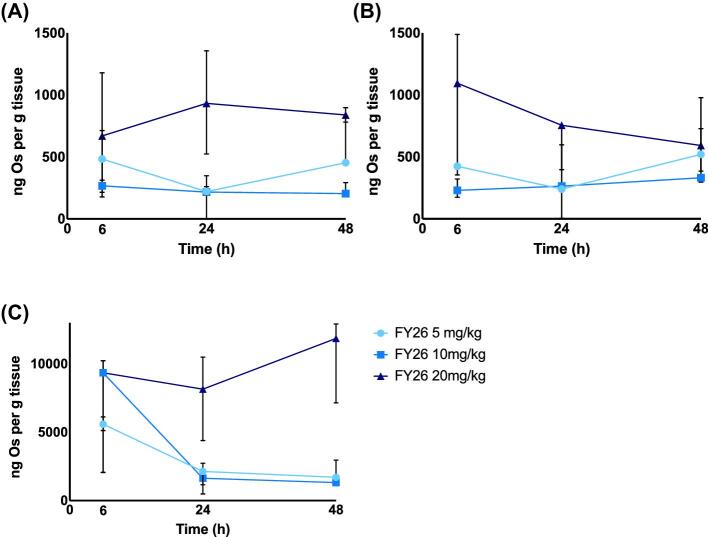
Dose-dependent kinetics of osmium concentrations in tumour and liver tissues from mice (*N* = 3): (A) left tumour; (B) right tumour; and (C) liver. All data points are median ± IQR.

Following the administration of FY26⋅Cl to Hepa1–6-*Per2-luc* tumour-bearing mice at ZT6, the tissue [Os] concentrations varied as a function of dose at 6 h, in the tumours in each flank and in liver (Fig. 7). Based on these data, FY26⋅Cl tumour uptake was nearly 10-fold less in both tumours as compared to liver at 6 h post-treatment.

The tumour [Os] levels remained almost constant over time in the mice dosed with 20 mg/kg, with similar values in the right and left flank tumours sampled at 6, 24, and 48 h after injection (Fig. 7A and B). In contrast, the [Os] in the tumours of both flanks decreased at 24 h and slightly increased at 48 h for both lower dose levels (10 and 5 mg/kg). In the liver, similar [Os] concentrations were found 6 h after dosing the mice with 20 or 10 mg/kg, while [Os] levels nearly halved in the mice treated with 5 mg/kg (Fig. 7C). Liver [Os] levels remained almost constant until 48 h for the mice treated with 20 mg/kg of FY26⋅Cl. In contrast, the liver [Os] concentrations decreased sharply at 24 h and remained stable until 48 h for the 10 and 5 mg/kg doses.

Taken together, these tissue pharmacokinetic data suggest that some uptake saturation mechanisms and efflux blockage for at least 48 h are reached both in the tumour and in the liver with a single ip dose of 20 mg/kg of FY26⋅Cl. The partial clearance of liver Os at 24 h in the mice dosed with 10 or 5 mg/kg could result in Os re-uptake by the tumours from the bloodstream, hence accounting for the slight tumour [Os] increase at 48 h.

All the treated mice lost some weight 24 and or 48 h post-dosing, while controls gained some weight over this time span. Nevertheless, body weight changes were <5% in all the mice, whatever the dose level and day of assessment. The limited sample size and low toxicity of a single dose precluded the demonstration of any dose–response relationship, using body weight changes as an endpoint. No changes in tumour volumes were found, as expected following a single FY26 dose.

## Discussion

Currently, the most widely administered drugs in cancer chemotherapy are platinum complexes (cisplatin, carboplatin, and oxaliplatin).^26^ Despite the clinical efficacy of these drugs, there is a need for new drugs with different mechanisms of action that can circumvent platinum resistance, widen the spectrum of activity, and reduce side effects.^27^

Organo-osmium anticancer complexes with PF_6_ anions have high crystal packing energies, and we previously reported that solubility can be improved up to 45-fold by switching to CF_3_SO_3_ as a counter anion.^23^ Here, we show that FY26⋅X solubility is vastly improved 10,448-fold by switching to a chloride anion. Such drastic solubility improvements eliminate the need for DMSO in biological assays and improve viability for clinical use. Furthermore, since the active component is the organo-osmium cation, the change in anion did not significantly alter its pharmacological activity.

Among other precious metals, complexes of Os(II), also a third row transition metal, and isoelectronic with Pt(IV), show promise. Here, we have studied the organo-osmium(II) half-sandwich complex FY26 ([Os^II^(ɳ^6^-*p*-cym)(PhAzPy-NMe_2_)I]^+^), a pseudo-octahedral complex in which the arene *p*-cymene occupies three coordination positions, the phenylazopyridine ligand is chelated, and a strongly bound iodide ligand completes the coordination sphere.^28^

FY26 is a relatively inert complex that does not readily hydrolyse even after 24 h incubation at 37°C in aqueous solution at pH 7.4, or react with DNA bases.^12^ However, radiolabelling studies with ^131^I have shown that the complex is activated in cancer cells and the iodide ligand is rapidly excreted.^16^ The activation of FY26 appears to involve attack on the azo bond of the chelated ligand by the abundant intracellular tripeptide glutathione, which weakens the Os–I bond.^17^ This complex appears to act as a metabolic inhibitor, inducing apoptotic cell death via ROS generation.^3,4^ The mechanism also involves the arrest of cycling cells in G1 phase, thus halting cellular proliferation.^12^

Here, a consistent yet moderate efficacy of the osmium complex FY26 was demonstrated in hepatocarcinoma-bearing mice, as indicated with an ∼60% tumour inhibition as compared to controls using the more soluble FY26⋅Cl. Indeed, both FY26⋅Cl and FY26⋅PF_6_ displayed some efficacy in this tumour model, both *in vitro* and *in vivo.*^22^ Reaching a cumulative dose of 60 mg/kg appeared to be necessary for FY26⋅Cl efficacy to become apparent.

Since the circadian clock regulates both cellular metabolism and the cell cycle,^6,7^ we sought whether circadian timing would moderate FY26 effects in this mouse hepatocarcinoma model, both in cell cultures and in tumour-bearing mice, and aimed at the determination of implications for optimization of treatment schedules.

The dosing time of FY26 significantly impacted on FY26 tolerability, as indicated by the loss in body weight of the tumour-bearing mice. The mice treated at ZT18 showed larger loss in body weight as compared to those treated at ZT6. This is consistent both with the current studies using FY26⋅PF_6_ or FY26⋅Cl and with prior experiments where FY26⋅PF_6_ had been administered at six ZTs in non-tumour-bearing mice.^22^

The blockbuster platinum complex oxaliplatin has largely benefitted from circadian research early on, at a time when its development as an anticancer drug had been halted for undue clinical toxicities.^29^ More specifically, the toxicities of oxaliplatin, like those of carboplatin and cisplatin, were halved or reduced even more through their administration in mice or rats during their nocturnal activity span.^30,31^ Interestingly, the antitumour efficacies of both cisplatin and oxaliplatin were enhanced following dosing at the time when both drugs were least toxic.^32,33^ Moreover, early afternoon delivery of oxaliplatin significantly improved tolerability and efficacy of oxaliplatin in metastatic colorectal cancer patients.^9,34^ Reduced glutathione plays a key role in the circadian detoxification mechanisms of the platinum complexes. Thus, the cytosolic concentrations of reduced glutathione more than doubled along the 24-h timescale in mouse liver with a statistically significant circadian rhythm.^35^ The 24-h mean of GSH concentrations was more than halved following the administration of l-BSO, an inhibitor of gamma-glutamylcysteine synthetase, a rate-limiting enzyme of GSH synthesis. Moreover, the GSH pattern became bimodal over the 24 h after l-BSO exposure. The rhythmic patterns in cisplatin toxicities closely matched those of GSH. Thus, the prominent circadian rhythm that characterized cisplatin^30,31^ was drastically altered in the BSO-treated mice, with a 5.7-fold reduction in average survival rate, and a shift in the period of cisplatin toxicity rhythm from 24 to 12 h.^36^ Reduced glutathione, however, appears to have a dual role for FY26 pharmacology; i.e. it can facilitate the bioactivation of FY26 into the corresponding aqua species, but also detoxify FY26 in cells. This may account for the difference of several hours regarding the circadian times of administration that optimize the tolerability of platinum complexes on the one hand and of FY26 on the other hand, both *in vitro* and *in vivo*.^22^^30,31^

In spite of the larger body weight loss, the mice receiving FY26⋅Cl at ZT18 had a larger tumour-weight reduction as compared to those injected at ZT6. The apparent increase in antitumour efficacy following dosing at the circadian time of poor tolerability strikingly differs from prior findings with other anticancer agents, including cisplatin and oxaliplatin. Thus, the dosing time of best efficacy coincided with that of best tolerability for 28 anticancer drugs.^6–8^ However, spontaneous regressions and regrowth characterized the experimental model we used here, thus indicating the occurrence of host immunological responses against the subcutaneous Hepa1–6 tumours, which were growing in genetically related yet non-syngeneic mice (C57Bl/6 instead of C57L/J).^35,37^ Our study suggests that treatment schedules should indeed aim to reduce the exposure of healthy tissues to FY26⋅Cl through drug dosing at ZT6, because (i) a significant tumour drug uptake was demonstrated at this time, (ii) a significant tumour inhibition was achieved in mice treated at this time with the more dose-intensive schedule (Experiment 3), and (iii) the optimization of drug tolerability was crucial for outcomes (Experiment 4).

## Conclusions

Here, we have demonstrated that FY26⋅Cl and FY26⋅PF_6_ are least toxic to the mice, following dosing near the middle of their resting span, during daytime. The circadian pattern in the FY26⋅Cl tolerability that was found here was similar to that demonstrated earlier for the less soluble FY26⋅PF_6_ formulation. The circadian optimization of tolerability appeared potentially useful, because FY26-induced antitumour efficacy required treatment with repeated administrations. Thus, good tolerability would favour compliance to the subchronic treatment that was needed to achieve efficacy. Since the tumour drug levels remained similar at 48 h post-dose, irrespective of dose level, we propose to enhance treatment efficacy through the daily delivery of low dose levels (i.e. 5–10 mg/kg) at ZT6, a high dose intensity schedule that could be automatically delivered using ‘intelligent’ nanoparticles.

## Data Availability

The data that support the findings in this study are available in the Warwick Research Archive Portal (WRAP) repository, http://wrap.warwick.ac.uk/145362/.
